# The genome sequence of the large burdock *Cheilosia*, *Cheilosia vulpina* (Meigen, 1822)

**DOI:** 10.12688/wellcomeopenres.17491.1

**Published:** 2021-12-17

**Authors:** Steven Falk, University of Oxford and Wytham Woods Genome Acquisition Lab, Natural History Museum Genome Acquisition Lab, Darwin Tree of Life Barcoding collective, Wellcome Sanger Institute Tree of Life programme, Wellcome Sanger Institute Scientific Operations: DNA Pipelines collective, Tree of Life Core Informatics collective, Darwin Tree of Life Consortium

**Affiliations:** 1 Independent Researcher, Kenilworth, UK

**Keywords:** Cheilosia vulpina, large burdock Cheilosia, genome sequence, chromosomal, Diptera

## Abstract

We present a genome assembly from an individual female *Cheilosia vulpina *(the large burdock *Cheilosia *or stocky blacklet; Arthropoda; Insecta; Diptera; Syriphidae). The genome sequence is 913 megabases in span. The majority of the assembly (98.81%) is scaffolded into sixchromosomal pseudomolecules, with the X sex chromosome assembled.

## Species taxonomy

Eukaryota; Metazoa; Ecdysozoa; Arthropoda; Hexapoda; Insecta; Pterygota; Neoptera; Endopterygota; Diptera; Brachycera; Muscomorpha; Syrphoidea; Syrphidae; Eristalinae; Rhingiini; Cheilosia; *Cheilosia vulpina* (Meigen, 1822) (NCBI:txid273409).

## Background

*Cheilosia vulpina* (the large burdock *Cheilosia* or the stocky blacklet) is a stocky, medium-large *Cheilosia* with outstanding hairs on the face. The males can be distinguished from other large hairy-faced *Cheilosia* by the longer body hairs, especially the scutellar marginals and hairs around the margins of the abdomen (much shorter in *C. lasiopa*) and pale bases to the tibiae (dark in the much longer-winged *C. variabilis*). The females have very noticeable 'fasciae' of pale hairs across the tergites and look like large, well-marked specimens of *C. proxima* or the scarcer *C. velutina* in the field (neither of which have outstanding facial hairs).

In the UK, *C. vulpina* is widespread though localised to southern England and seems to be most frequent in calcareous grasslands. It has been reared from the roots of greater and lesser burdocks in Germany (the probable foodplants in Britain) and also globe artichoke outside of the UK. There are two generations a year, and the spring brood averages larger and hairier than the summer one. The summer generation was once regarded as a separate species, *C. conops*. Adults are particularly keen on the flowers of umbellifers, including cow parsley in spring, and angelica, wild parsnip, and upright hedge-parsley in summer.

## Genome sequence report

**Figure 1.**
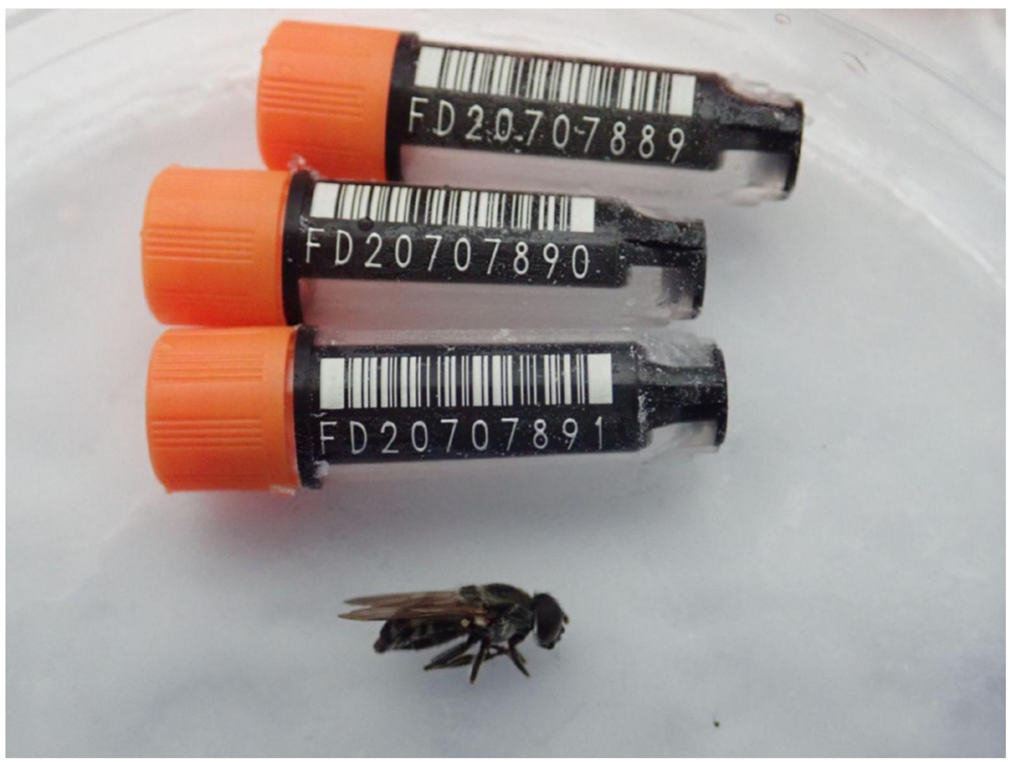
Image of the sequenced idCheVulp2 specimen taken during preservation and processing.

The genome was sequenced from a single female *C. vulpina* ( Figure 1) collected from Wytham Woods, Oxfordshire (biological vice-county: Berkshire), UK (latitude 51.77, longitude -1.331). A total of 51-fold coverage in Pacific Biosciences single-molecule long reads and 120-fold coverage in 10X Genomics read clouds were generated. Primary assembly contigs were scaffolded with chromosome conformation Hi-C data. Manual assembly curation corrected 11 missing/misjoins, reducing the assembly length by 0.11% and the scaffold number by 33.33%, and increasing the scaffold N50 by 52.44%.

**Figure 2.**
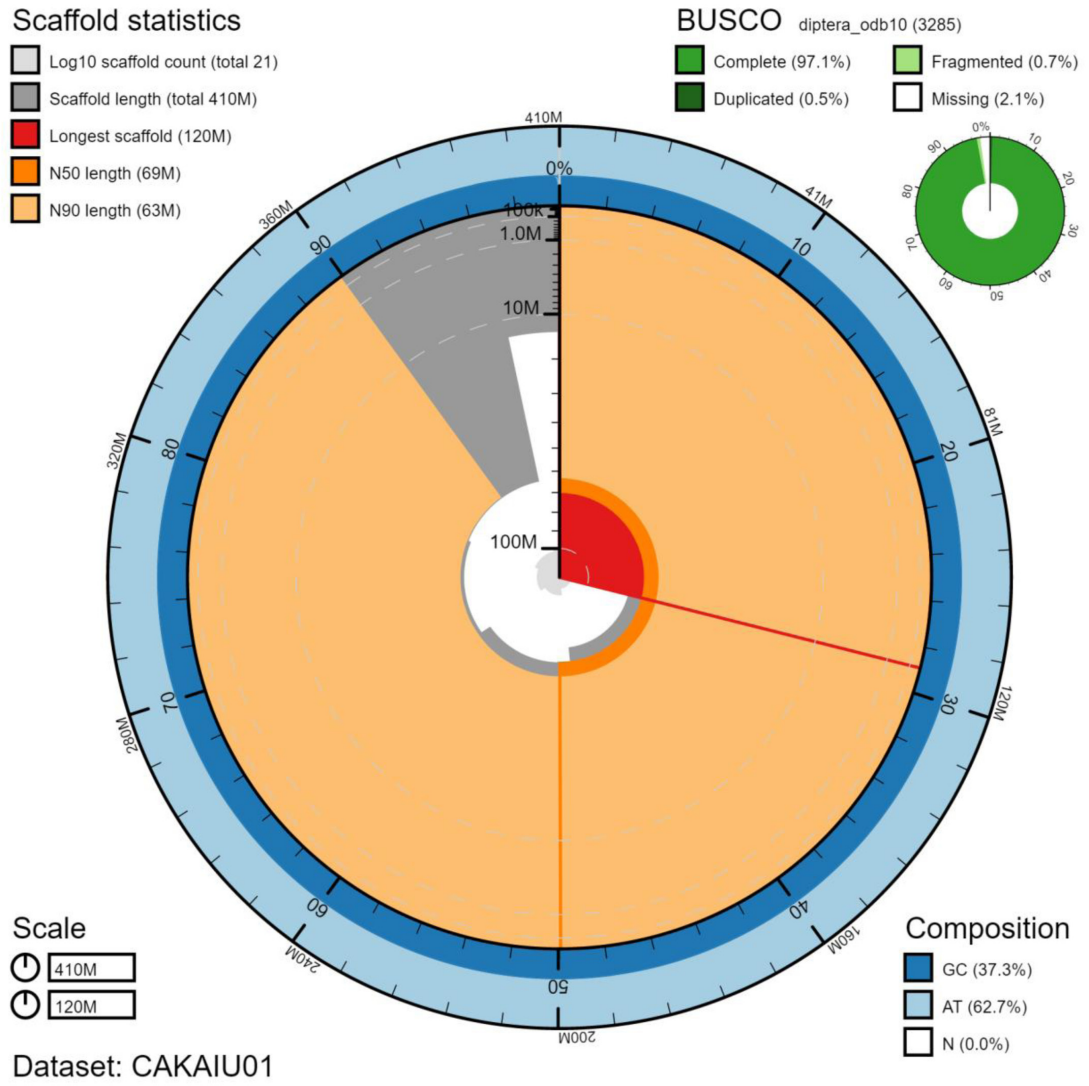
Genome assembly of *Cheilosia vulpina*, idCheVulp2.1: metrics. The BlobToolKit Snailplot shows N50 metrics and BUSCO gene completeness. The main plot is divided into 1,000 size-ordered bins around the circumference with each bin representing 0.1% of the 405,383,207 bp assembly. The distribution of chromosome lengths is shown in dark grey with the plot radius scaled to the longest chromosome present in the assembly (117,531,788 bp, shown in red). Orange and pale-orange arcs show the N50 and N90 chromosome lengths (69,418,634 and 63,252,922 bp), respectively. The pale grey spiral shows the cumulative chromosome count on a log scale with white scale lines showing successive orders of magnitude. The blue and pale-blue area around the outside of the plot shows the distribution of GC, AT and N percentages in the same bins as the inner plot. A summary of complete, fragmented, duplicated and missing BUSCO genes in the diptera_odb10 set is shown in the top right. An interactive version of this figure is available at https://blobtoolkit.genomehubs.org/view/idCheVulp2.1/dataset/CAKAIU01/snail.

The final assembly has a total length of 405 Mb in 20 sequence scaffolds with a scaffold N50 of 69.4 Mb ( Table 1). The majority, 99.92%, of the assembly sequence was assigned to 6 chromosomal-level scaffolds, representing 5 autosomes (numbered by sequence length), and the X sex chromosome ( Figure 2– Figure 5; Table 2). The assembly has a BUSCO completeness of 97.1% (single 96.7%, duplicated 0.5%) using the diptera_odb10 reference set. While not fully phased, the assembly deposited is of one haplotype. Contigs corresponding to the second haplotype have also been deposited.

**Table 1.** Genome data for *Cheilosia vulpina*, idCheVulp2.1.

*Project accession data*	
Assembly identifier	idCheVulp2.1
Species	*Cheilosia vulpina*
Specimen	idCheVulp2 (genome assembly, Hi-C); idCheVulp1 (Hi-C)
NCBI taxonomy ID	273409
BioProject	PRJEB46307
BioSample ID	SAMEA7746587
Isolate information	Female, thorax, head (idCheVulp2); Unknown sex, head (idCheVulp1)
*Raw data accessions*	
PacificBiosciences SEQUEL II	ERR6807994
10X Genomics Illumina	ERR6688460-ERR6688463
Hi-C Illumina	ERR6688459, ERR6688464
*Genome assembly*	
Assembly accession	GCA_916610125.1
*Accession of alternate haplotype*	GCA_916610195.1
Span (Mb)	405
Number of contigs	30
Contig N50 length (Mb)	45.5
Number of scaffolds	20
Scaffold N50 length (Mb)	69.4
Longest scaffold (Mb)	117.5
BUSCO * genome score	C:97.1%[S:96.7%,D:0.5%], F:0.7%,M:2.1%,n:3285

*BUSCO scores based on the diptera_odb10 BUSCO set using v5.1.2. C= complete [S= single copy, D=duplicated], F=fragmented, M=missing, n=number of orthologues in comparison. A full set of BUSCO scores is available at https://blobtoolkit.genomehubs.org/view/idCheVulp2.1/dataset/CAKAIU01/busco.

**Figure 3.**
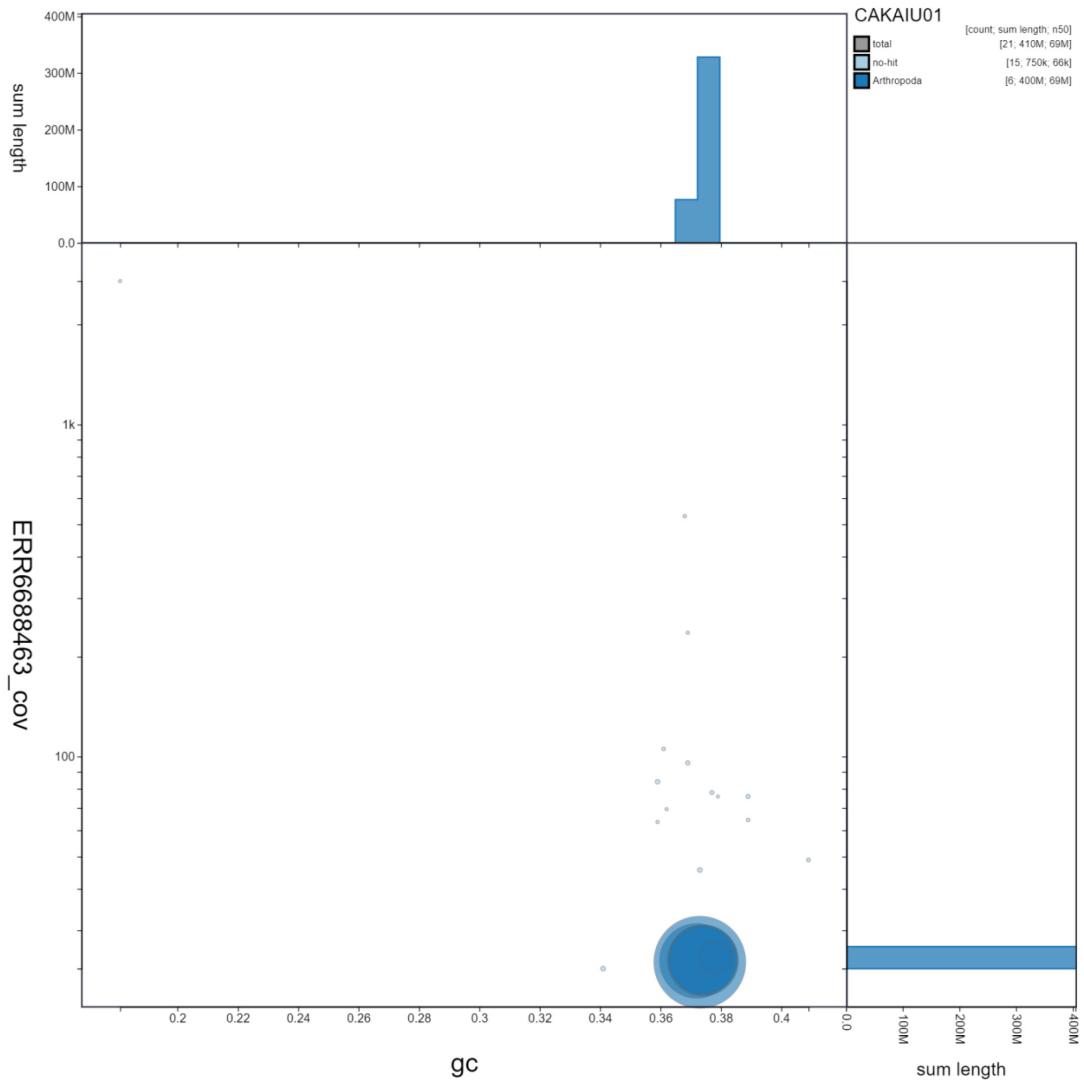
Genome assembly of *Cheilosia vulpina*, idCheVulp2.1: GC coverage. BlobToolKit GC-coverage plot. Scaffolds are coloured by phylum. Circles are sized in proportion to scaffold length. Histograms show the distribution of scaffold length sum along each axis. An interactive version of this figure is available at https://blobtoolkit.genomehubs.org/view/idCheVulp2.1/dataset/CAKAIU01/blob.

**Figure 4.**
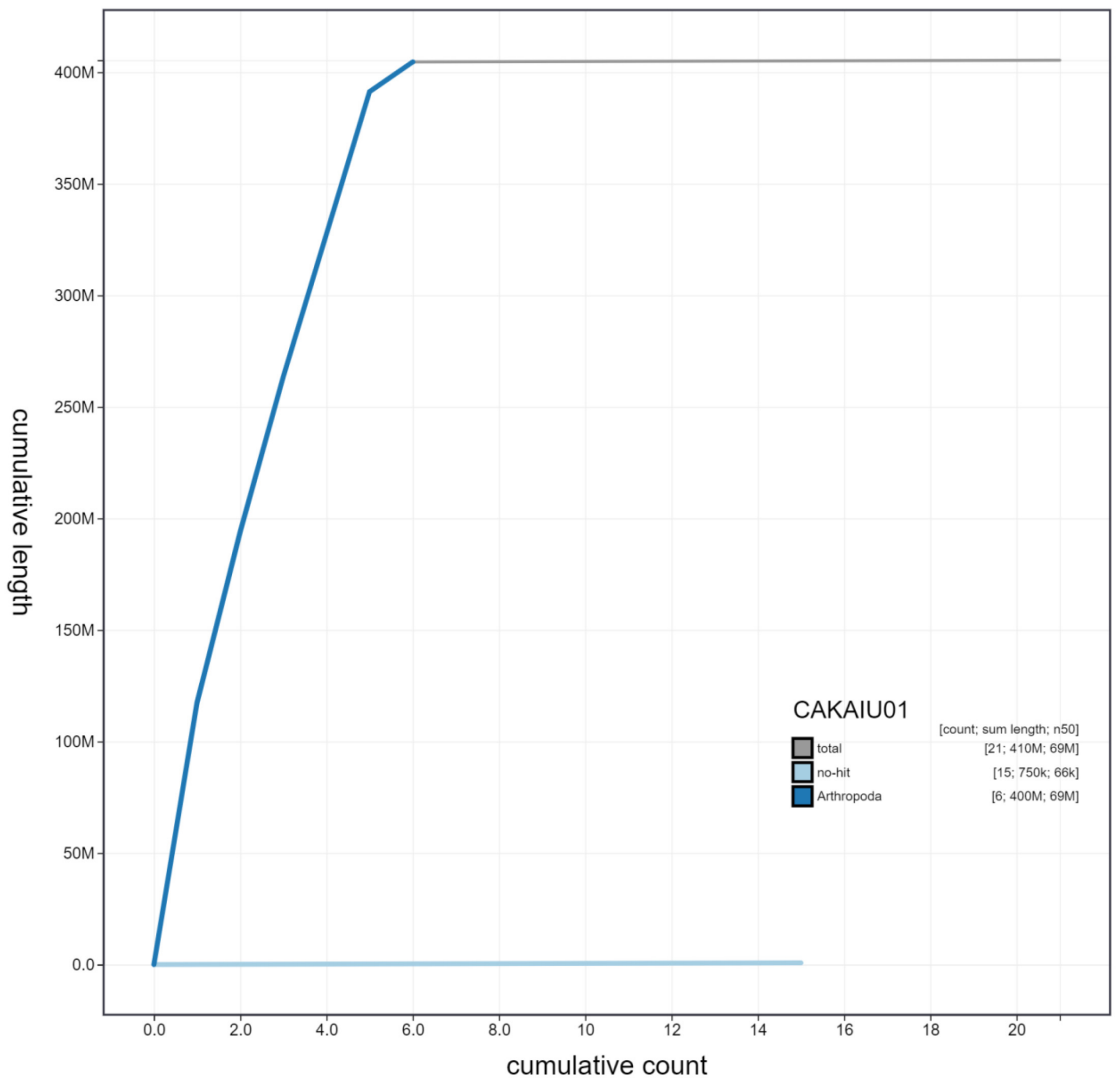
Genome assembly of *Cheilosia vulpina*, idCheVulp2.1: cumulative sequence. BlobToolKit cumulative sequence plot. The grey line shows cumulative length for all scaffolds. Coloured lines show cumulative lengths of scaffolds assigned to each phylum using the buscogenes taxrule. An interactive version of this figure is available at https://blobtoolkit.genomehubs.org/view/idCheVulp2.1/dataset/CAKAIU01/cumulative.

**Figure 5.**
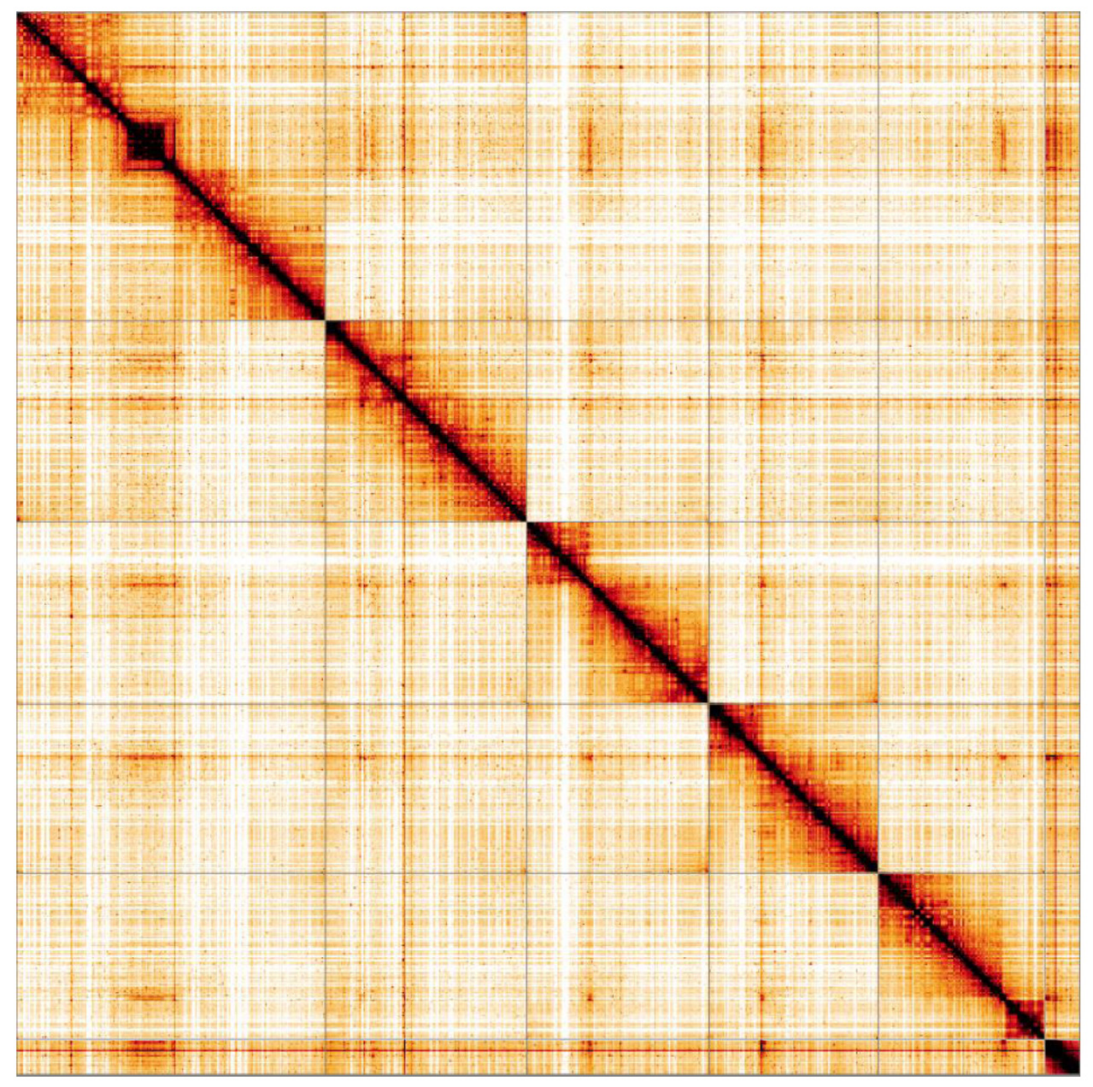
Genome assembly of *Cheilosia vulpina*, idCheVulp2.1: Hi-C contact map. Hi-C contact map of the icCheVulp2.1 assembly, visualised in HiGlass. Chromosomes are given in order of size from left to right and top to bottom.

**Table 2.** Chromosomal pseudomolecules in the genome assembly of *Cheilosia vulpina*, idCheVulp2.1.

INSDC accession	Chromosome	Size (Mb)	GC%
OU744276.1	1	117.53	37.3
OU744277.1	2	76.59	37.2
OU744278.1	3	69.42	37.4
OU744279.1	4	64.49	37.4
OU744280.1	5	63.25	37.4
OU744281.1	X	13.34	37.8
OU744282.1	MT	0.02	18.1
-	Unplaced	0.74	36.9

## Methods

### Sample acquisition and nucleic acid extraction

A female *C. vulpina* (idCheVulp2) and a second *C. vulpina* of unknown sex (idCheVulp1) were collected from Wytham Woods, Oxfordshire (biological vice-county: Berkshire), UK (latitude 51.77, longitude -1.331) by Steven Falk, independent researcher, who also identified the specimens. The specimens were collected using a net and snap-frozen on dry ice.

DNA was extracted at the Tree of Life laboratory, Wellcome Sanger Institute. The idCheVulp2 sample was weighed and dissected on dry ice with tissue set aside for Hi-C sequencing. Abdomen tissue was disrupted using a Nippi Powermasher fitted with a BioMasher pestle. Fragment size analysis of 0.01–0.5 ng of DNA was then performed using an Agilent FemtoPulse. High molecular weight (HMW) DNA was extracted using the Qiagen MagAttract HMW DNA extraction kit. Low molecular weight DNA was removed from a 200-ng aliquot of extracted DNA using 0.8X AMpure XP purification kit prior to 10X Chromium sequencing; a minimum of 50 ng DNA was submitted for 10X sequencing. HMW DNA was sheared into an average fragment size between 12-20 kb in a Megaruptor 3 system with speed setting 30. Sheared DNA was purified by solid-phase reversible immobilisation using AMPure PB beads with a 1.8X ratio of beads to sample to remove the shorter fragments and concentrate the DNA sample. The concentration of the sheared and purified DNA was assessed using a Nanodrop spectrophotometer and Qubit Fluorometer and Qubit dsDNA High Sensitivity Assay kit. Fragment size distribution was evaluated by running the sample on the FemtoPulse system.

### Sequencing

Pacific Biosciences HiFi circular consensus and 10X Genomics Chromium read cloud sequencing libraries were constructed according to the manufacturers’ instructions. Sequencing was performed by the Scientific Operations core at the Wellcome Sanger Institute on Pacific Biosciences SEQUEL II and Illumina NovaSeq 6000 instruments. Hi-C data were generated from head tissue of idChrBici1 and idCheVulp2 using the Arima Hi-C+ kit and sequenced on a NovaSeq 6000 instrument.

### Genome assembly

Assembly was carried out with Hifiasm ( Cheng *et al.*, 2021); haplotypic duplication was identified and removed with purge_dups ( Guan *et al.*, 2020). One round of polishing was performed by aligning 10X Genomics read data to the assembly with longranger align, calling variants with freebayes ( Garrison & Marth, 2012). The assembly was then scaffolded with Hi-C data ( Rao *et al.*, 2014) using SALSA2 ( Ghurye *et al.*, 2019). The assembly was checked for contamination and corrected using the gEVAL system ( Chow *et al.*, 2016) as described previously ( Howe *et al.*, 2021). Manual curation was performed using gEVAL, HiGlass ( Kerpedjiev *et al.*, 2018) and Pretext. The mitochondrial genome was assembled using MitoHiFi ( Uliano-Silva *et al.*, 2021), with annotation performed using MitoFinder. The genome was analysed and BUSCO scores generated within the BlobToolKit environment ( Challis *et al.*, 2020). Table 3 contains a list of all software tool versions used, where appropriate.

**Table 3.** Software tools used.

Software tool	Version	Source
Hifiasm	0.15.3-r339	Cheng *et al.*, 2021
purge_dups	1.2.3	Guan *et al.*, 2020
SALSA2	2.2	Ghurye *et al.*, 2019
longranger align	2.2.2	https://support.10xgenomics.com/genome-exome/ software/pipelines/latest/advanced/other-pipelines
freebayes	1.3.1-17-gaa2ace8	Garrison & Marth, 2012
MitoHiFi	2.0	Uliano-Silva *et al.*, 2021
HiGlass	1.11.6	Kerpedjiev *et al.*, 2018
PretextView	0.2.x	https://github.com/wtsi-hpag/PretextView
BlobToolKit	2.6.2	Challis *et al.*, 2020

### Ethics/compliance issues

The materials that have contributed to this genome note have been supplied by a Darwin Tree of Life Partner. The submission of materials by a Darwin Tree of Life Partner is subject to the Darwin Tree of Life Project Sampling Code of Practice. By agreeing with and signing up to the Sampling Code of Practice, the Darwin Tree of Life Partner agrees they will meet the legal and ethical requirements and standards set out within this document in respect of all samples acquired for, and supplied to, the Darwin Tree of Life Project. Each transfer of samples is further undertaken according to a Research Collaboration Agreement or Material Transfer Agreement entered into by the Darwin Tree of Life Partner, Genome Research Limited (operating as the Wellcome Sanger Institute), and in some circumstances other Darwin Tree of Life collaborators.

## Data availability

European Nucleotide Archive: Cheilosia vulpina (large burdock Cheilosia). Accession number PRJEB46307: https://www.ebi.ac.uk/ena/browser/view/PRJEB46307.

The genome sequence is released openly for reuse. The *C. vulpina* genome sequencing initiative is part of the Darwin Tree of Life (DToL) project. All raw sequence data and the assembly have been deposited in INSDC databases. The genome will be annotated and presented through the Ensembl pipeline at the European Bioinformatics Institute. Raw data and assembly accession identifiers are reported in Table 1.

## Author information

Members of the University of Oxford and Wytham Woods Genome Acquisition Lab are listed here: https://doi.org/10.5281/zenodo.5746938.

Members of the Darwin Tree of Life Barcoding collective are listed here: https://doi.org/10.5281/zenodo.5744972.

Members of the Wellcome Sanger Institute Tree of Life programme are listed here: https://doi.org/10.5281/zenodo.5744840.

Members of Wellcome Sanger Institute Scientific Operations: DNA Pipelines collective are listed here: https://doi.org/10.5281/zenodo.5746904.

Members of the Tree of Life Core Informatics collective are listed here: https://doi.org/10.5281/zenodo.5743293.

Members of the Darwin Tree of Life Consortium are listed here: https://doi.org/10.5281/zenodo.5638618.

## References

[ref-2] Challis R Richards E Rajan J : BlobToolKit - Interactive Quality Assessment of Genome Assemblies. *G3 (Bethesda).* 2020;10(4):1361–74. 10.1534/g3.119.400908 32071071 PMC7144090

[ref-3] Cheng H Concepcion GT Feng X : Haplotype-Resolved *de Novo* Assembly Using Phased Assembly Graphs with Hifiasm. *Nat Methods.* 2021;18(2):170–75. 10.1038/s41592-020-01056-5 33526886 PMC7961889

[ref-4] Chow W Brugger K Caccamo M : gEVAL - a web-based browser for evaluating genome assemblies. *Bioinformatics.* 2016;32(16):2508–10. 10.1093/bioinformatics/btw159 27153597 PMC4978925

[ref-5] Garrison E Marth G : Haplotype-Based Variant Detection from Short-Read Sequencing. 2012; arXiv: 1207.3907. Reference Source

[ref-6] Ghurye J Rhie A Walenz BP : Integrating Hi-C Links with Assembly Graphs for Chromosome-Scale Assembly. *PLoS Comput Biol.* 2019;15(8):e1007273. 10.1371/journal.pcbi.1007273 31433799 PMC6719893

[ref-7] Guan D McCarthy SA Wood J : Identifying and Removing Haplotypic Duplication in Primary Genome Assemblies. *Bioinformatics.* 2020;36(9):2896–98. 10.1093/bioinformatics/btaa025 31971576 PMC7203741

[ref-8] Howe K Chow W Collins J : Significantly Improving the Quality of Genome Assemblies through Curation. *GigaScience.* 2021;10(1):giaa153. 10.1093/gigascience/giaa153 33420778 PMC7794651

[ref-9] Kerpedjiev P Abdennur N Lekschas F : HiGlass: Web-Based Visual Exploration and Analysis of Genome Interaction Maps. *Genome Biol.* 2018;19(1):125. 10.1186/s13059-018-1486-1 30143029 PMC6109259

[ref-12] Rao SSP Huntley MH Durand NC : A 3D Map of the Human Genome at Kilobase Resolution Reveals Principles of Chromatin Looping. *Cell.* 2014;159(7):1665–80. 10.1016/j.cell.2014.11.021 25497547 PMC5635824

[ref-16] Uliano-Silva M Nunes JGF Krasheninnikova K : marcelauliano/MitoHiFi: mitohifi_v2.0. 2021. 10.5281/zenodo.5205678

