# Supplementary material for: The genome sequence of the large burdock Cheilosia, Cheilosia vulpina (Meigen, 1822)
Source: Wellcome Open Res. 2021 Dec 17;6:351. [Version 1] doi: 10.12688/wellcomeopenres.17491.1 (PMC9652505; doi:10.12688/wellcomeopenres.17491.1)
Supplement: Peer review history

# Peer review: Reviewer response for version 1

**DOI:** 10.21956/wellcomeopenres.19341.r53053

**Published:** 2022-11-11

**Authors:** Ljiljana Šašić Zorić

**Affiliations:** 1 BioSense Institute, Novi Sad, Serbia

“The genome sequence of the large burdock Cheilosia, Cheilosia vulpina (Meigen, 1822)” is presented in the form of a technical report on the genome assembly of hoverfly species Cheilosia vulpina. The article is an important contribution to further research on hoverflies being known as the second most important pollinators among insects. It describes technical aspects of genome assembly clearly and concisely. The applied approach includes PacBio and 10X sequencing data, with scaffolding using Hi-C data. The methodology is suitable and overall well described, although some minor inconsistencies should be resolved before final publication.

 Suggestions for revision:

 Abstract

- The genome assembly total length and per cent of the assembly sequence assigned to 6 chromosomal-level scaffolds (913M and 98.81%) are not in agreement with the main text (405M and 99.92%).
- “sixchromosomal” should be written separately “six chromosomal”

Genome sequence report

- It would be good if the caption for Figure 1 refers to the name of the species, not only ID. I would suggest editing the figure caption in the following way: “Image of the sequenced Cheilosia vulpina (idCheVulp2) specimen…”
- In the second paragraph, it is stated that the final assembly has a total length of 405 Mb in 20 sequence scaffolds. The same is stated in Table 1. However, based on Figure 2 and results available at https://blobtoolkit.genomehubs.org/view/idCheVulp2.1/dataset/CAKAIU01/snail it seems that there are 21 scaffolds.
- Figure 2 is a bit different compared to the interactive version on the link https://blobtoolkit.genomehubs.org/view/idCheVulp2.1/dataset/CAKAIU01/snail (scale in the interactive version is set to 405 and 118M, while in the text is 410M and 120M). Please check this.
- Figure 3 needs some technical corrections such as first letter capitalization in the names of axes and gc should also be capitalized

Methods

- It is stated that the gender of specimen idCheVulp1 is not known. What is the reason not to determine gender? Is the specimen damaged? Please state this.
- There is inconsistency in the text regarding the use of idCheVulp1 specimen in analysis. Although based on Table 1 it is used for Hi-C, only for idCheVulp2 sample is stated that tissue was set aside for Hi-C sequencing. Additionally, it is written that Hi-C data were generated from the head tissue of idChrBici1 and idCheVulp2, but there is not any additional information for specimen idChrBici1.
- There is no information on parameters used in data analysis. If the default values were used please state that clearly or, in case the data analysis follows the procedure previously described in detail, please refer to the source.

Are sufficient details of methods and materials provided to allow replication by others?

Partly

Is the rationale for creating the dataset(s) clearly described?

Yes

Are the datasets clearly presented in a useable and accessible format?

Yes

Are the protocols appropriate and is the work technically sound?

Yes

Reviewer Expertise:

Population genetics and molecular taxonomy

I confirm that I have read this submission and believe that I have an appropriate level of expertise to confirm that it is of an acceptable scientific standard.

# Peer review: Reviewer response for version 1

**DOI:** 10.21956/wellcomeopenres.19341.r47741

**Published:** 2022-01-18

**Authors:** Nallur B. Ramachandra, Koushik Ponnanna

**Affiliations:** 1 Department of Studies in Genetics and Genomics, University of Mysore, Mysuru, Karnataka, India; 2 University of Mysore, Mysore, India

Genome sequencing of plants and animals, including humans, is generating enormous data to understand the biology, structure, and function of the organism. One such event is this manuscript wherein a single female of the large burdock *Cheilosia vulpina* (Meigen, 1822) is being reported.

 The methodology followed for DNA isolation, sequencing, and assembling the genome is well organized. The organelle DNA is also taken into account with necessary contamination removal. This kind of information certainly adds to the genome resources of insects.

 However, we encountered the following concerns:

1. Throughout the manuscript, it is mentioned that a single female is sequenced. Is it impregnated or virgin? If it is impregnated, how do you account for possible male-specific genomic content?
2. Information about the available genome studies about *C. vulpina* is lacking in this manuscript.
3. Comparison of this assembly with the already available genome would have helped in appreciating the accuracy achieved.
4. Long read sequencing generally introduces sequencing errors. How was this taken care of in this study?

Overall, the data will definitely add to the existing genomic resources for *C. vulpina* which otherwise lacks a rich resource necessary for comparative studies.

Are sufficient details of methods and materials provided to allow replication by others?

Partly

Is the rationale for creating the dataset(s) clearly described?

Yes

Are the datasets clearly presented in a useable and accessible format?

Yes

Are the protocols appropriate and is the work technically sound?

Yes

Reviewer Expertise:

Genomics of Drosophila and Humans

We confirm that we have read this submission and believe that we have an appropriate level of expertise to confirm that it is of an acceptable scientific standard.
